# A delayed diagnosis of atypical immune dysregulation, polyendocrinopathy, enteropathy, X-linked (IPEX) syndrome: A case report

**DOI:** 10.1097/MD.0000000000025174

**Published:** 2021-03-26

**Authors:** Ying Zhang, Hanmin Liu, Tao Ai, Wanmin Xia, Tingting Chen, Lei Zhang, Xiulan Luo, Yaping Duan

**Affiliations:** a Key Laboratory of Birth Defects and Related Diseases of Women and Children (Sichuan University), Ministry of Education, West China Second University Hospital, Sichuan University, Chengdu, Sichuan; b Chengdu Women's and Children's Central Hospital; c West China University Hospital, Sichuan University.

**Keywords:** forkhead box protein 3, immune dysregulation, polyendocrinopathy, enteropathy, X-linked syndrome

## Abstract

**Introduction:**

Immune dysregulation, polyendocrinopathy, enteropathy, and X-linked (IPEX) syndrome is a rare monogenic autoimmune disease, which is caused by mutations in the forkhead box protein 3 gene, can affect various systems. The typical clinical manifestations of IPEX are enteropathy, type 1 diabetes mellitus, and skin diseases. However, some atypical phenotypes can easily be misdiagnosed clinically.

**Patient concerns:**

A 9-year-and-7-month old patient suffered from recurrent wheezing, hematochezia, and eczematous dermatitis at the age of six months, but did not have any manifestations of autoimmune endocrinopathy. The patient was treated with glucocorticoids for more than six years, and he developed bronchiectasis.

**Diagnosis:**

Whole exome sequencing revealed a hemizygous pathogenic mutation c.1010G>A, p. (Arg337Gln) in Forkhead box protein 3 gene (NM_014009.3).

**Interventions:**

The patient was treated with oral mycophenolate mofetil combined with inhaled budesonide formoterol for six months after diagnosis.

**Outcomes:**

The respiratory symptoms of the patient seemed to be controlled but eczematous dermatitis progressed, which led the patient to give up the treatment.

**Conclusion:**

Early diagnosis and treatment of IPEX are crucial. Lung injury may be a major problem in the later stages of atypical IPEX, and mycophenolate mofetil seems to control the respiratory symptoms, but could induce significant skin side effects.

## 1 Introduction

Immune dysregulation, polyendocrinopathy, enteropathy, and X-linked (IPEX) syndrome is a rare, often fatal, monogenic autoimmune disease that affects various systems. IPEX is caused by mutations in forkhead box protein 3 gene (*FOXP3*), which was first identified in 2000. Over 70 *FOXP3* mutations have been identified in patients with IPEX.^[1]^ Forkhead box protein 3 (FOXP3) has emerged as a key regulator of immune tolerance by virtue of its function as a master switch factor involved in the differentiation of regulatory T (Treg) cells.^[2]^ The typical clinical manifestations of IPEX are early-onset enteropathy, type 1 diabetes mellitus, and skin diseases. However, there are many different clinical manifestations of IPEX, and there is no clear genotype-phenotype correlation.^[3]^

## 2 Patient concerns

The patient was referred to the hospital at the age of 9 years and 7 months due to a 3-month history of cough with sputum and wheezing, which was more obvious at night and resulted in sleeplessness and hypoxia. The patient was born at term with a birth weight of 3300 g after an uneventful pregnancy and had no special family history. He developed recurrent wheezing from the age of six months and was diagnosed with asthma and started regular treatment at the age of 3 years. However, his condition was not controlled even with a high dose of inhaled corticosteroids.

Meanwhile, the patient began to experience recurrent hematochezia at the age of six months after introducing formula milk, accompanied by intermittent abdominal pain and vomiting. At the age of 4 years, he was diagnosed with allergic enteritis. Following a short period of remission after taking low-dose prednisone (1–2 mg/kg/day), the patient's illness recurred, and he was diagnosed with ulcerative colitis and food protein-mediated enterocolitis at seven years of age and started taking mesalazine, which seemed to control his digestive symptoms.

The patient also developed allergic rhinitis, allergic conjunctivitis, and eczema symptoms within the first year of life, which are still ongoing. He did not receive the vaccine as planned, and had chicken pox and hand, foot, and mouth disease in infancy. He was allergic to cefoxitin, piperacillin sulbactam, cefotaxime, and cefoperazone sulbactam, all of which presented as immediate generalized urticaria many days or course after the first infusion.

## 3 Clinic findings

On admission, he was afebrile and had a normal heart rate, blood pressure, and oxygen saturation in ambient air. However, he was shorter (124.0 cm, below the second percentile for age) and lighter (25.0 kg, below the first percentile for age) than normal children of the same age and sex. There were patches of lichenoid eczema on his trunk and limbs, but there were no palpable superficial lymph nodes. The fingers of the patient were clubbed. His respiration was labored despite a normal respiratory rate, and moderate wheezing and moist rales could be auscultated in bilateral lungs. No obvious abnormalities were found on examination of the heart, abdomen, and nervous systems.

The patient's routine blood test results were normal, except for eosinophil count and proportion, which could be masked by significant elevation of white blood cells (Table 1). The patient's stool and urine routine test, liver enzyme, serum albumin, serum electrolytes, renal function, and random blood glucose were normal. His serum IgG and IgM concentrations were also within the normal ranges, while IgA was consistently slightly below the normal range (Table 2), and IgE levels were significantly higher than normal (750 IU/mL, reference range 20–200 IU/mL). His proportion of CD3+, CD4+, CD8+, NK, and B cells was 80.2% (55–78%), 12.52% (23–53%), 58.08% (19–34%), 7.2% (14–26%), and 0.88% (10–31%), and CD4/CD8 ratio was 0.22. Autoantibodies including Anti-nRNP/Sm antibody, anti-SM antibody, anti-SS-A antibody, anti-Ro-52 antibody, anti-SS-B antibody, anti-SCl-70 antibody, anti-PM-Scl antibody, anti-JO-1 antibody, anti-CENP-B antibody, anti-PCNA antibody, anti-dsDNA antibody, anti-nucleosome antibody, anti-histone antibody, anti-Ribosomal P protein antibody, anti-AMA-M2 antibody were all negative. His levels of thyroid stimulating hormone, triiodothyronine, total thyroxine, free triiodothyronine, and free thyroxine were 4.373 (0.64–6.27) IU/mL, 1.13 (0.84–1.95) ng/mL, 10.5 (5.7–12.5) ug/dL, 4.27 (2.38–4.68) pg/mL, and 1.9 (1.03–1.59) ng/mL, respectively. Sputum culture showed growth of *Haemophilus influenzae*, and his tuberculosis γ-interferon test was negative.

**Table 1:** Blood routine results of hospitalisation.

Date	WBC (G/L)	Neutrophil (%)	Eosinophil (%)	Hemoglobin (g/L)	Thrombocyte (G/L)	CRP (mg/L)
2019-09-20	15.3 (3.6–9.7)	75.7 (23.6–75.0)	1.4 (0.0–6.8)	150 (110–146)	180 (100–450)	11 (0–10)
2019-10-02	38.11 (3.6–9.7)	85.5 (23.6–75.0)	0.5 (0.0–6.8)	148 (110–146)	193 (100–450)	39 (0–10)
2020-03-23	11.02 (3.6–9.7)	58.5 (23.6–75.0)	10.3 (0.0–6.8)	128 (110–146)	249 (100–450)	1 (0–10)

CRP = C-reaction protein, WBC = white blood cell.

**Table 2:** Serum immunoglobulin levels.

Date	IgG (g/L)	IgM (g/L)	IgA (g/L)	C3 (g/L)	C4 (g/L)
2011-8-14	10.67 (8–16)	0.96 (0.5–2.2)	0.23 (0.7–3.3)	0.99 (0.8–1.6)	0.14 (0.15–0.4)
2017-7-17	13.20 (8–16)	0.57 (0.5–2.2)	0.32 (0.7–3.3)	1.27 (0.8–1.6)	0.30 (0.15–0.4)
2020-3-23	10.33 (8–16)	0.51 (0.5–2.2)	0.20 (0.7–3.3)	1.19 (0.8–1.6)	0.17 (0.15–0.4)

**Figure 1:**
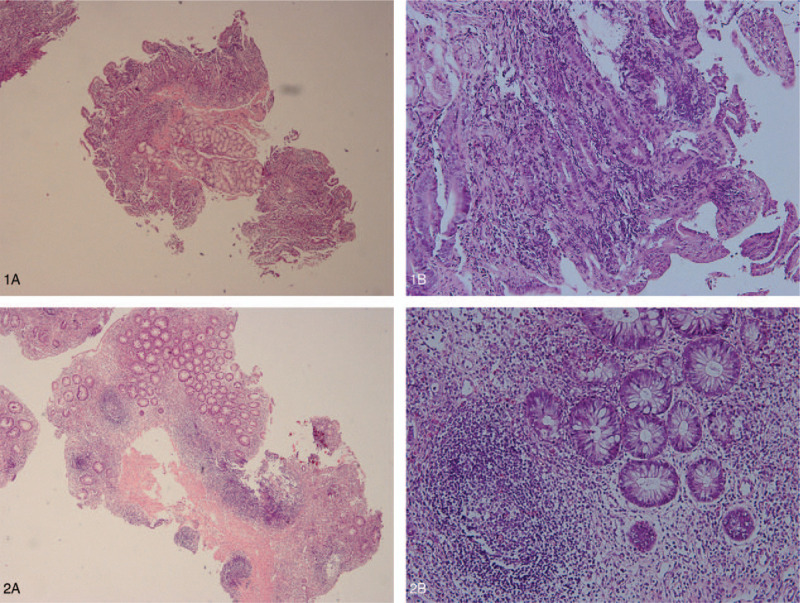
Pathological specimens stained with hematoxylin and eosin. 1, Pathologic images of the duodenal bulb; 2, Pathologic images of the sigmoid colon (A, magnification = 40x; B, magnification = 200 x).

Colonoscopy at 3 years of age demonstrated moderate to severe colonic proctitis, and pathological examination showed many eosinophils and a few lymphocytes and plasma cells in the intestinal mucosal stroma. Gastroscopy and colonoscopy at seven years of age demonstrated chronic superficial gastritis with flat erosion, duodenitis, and unconfirmed ulcerative colitis. His pathological examination demonstrated severe chronic inflammation of the mucous membrane, accompanied by erosion and ulcer formation, visible crypt inflammation and formation of crypt abscess, partial decrease or disappearance of glands, decreased secretion of mucus in some glands, multiple lymphoproliferative lesions in the sigmoid colon, light to moderate chronic inflammation of the mucosa with erosion, and a small amount of eosinophil infiltration in the stroma (<10/high-power field) of the duodenal bulb. Light to moderate chronic inflammation with erosion and much greater eosinophil infiltration in the stroma (50–100/ high power field) were observed in the descending duodenum (Fig. 1).

**Figure 2:**
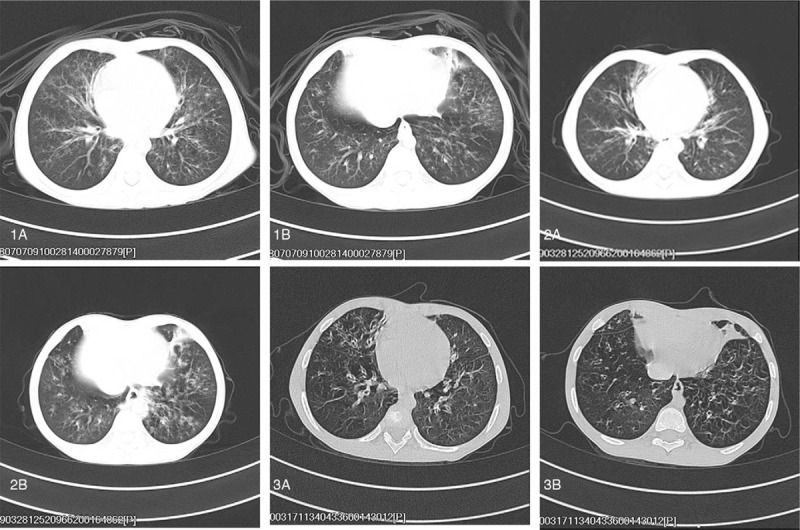
Chest high resolution computed tomography (HRCT): July 1, 2018: bilateral lung pneumonia with partial mild bronchiectasis. April 2, 2019: bronchiectasis was seen in the bilateral lungs. March 3, 2020: emphysema associated with bronchiectasis in the bilateral lungs.

The patient's pulmonary imaging findings showed gradually worsening bronchiectasis (Fig. 2), and the forced vital capacity (FVC), forced expiratory volume in one second, Forced expiratory volume in 1 second/FVC, forced expiratory flow at 50% and 75% of FVC, and maximum mid-expiratory flow of forced ventilation were significantly lower than normal (Table 3), indicating severe mixed ventilation dysfunction. The detection of pulmonary diffusing function demonstrated that the diffusion capacity for carbon monoxide in the lung was 83.1%, total lung capacity was 80.8%, residual volume was 168.25%, and residual volume/total lung capacity was 45.47%, which suggested that the pulmonary diffusing capacity was normal.

**Table 3:** Forced ventilation of lung function.

Date	FVC (%pre)	FEV1 (%pre)	FEV1/FVC (%pre)	FEF50 (%pre)	FEF75 (%pre)	MMEF (%pre)
2016-04-07	66.4	57.9	86.2	31.5	20.6	25.8
2017-10-26	66.1	57.7	86.3	33.3	29.3	32.2
2018-09-07	61.1	43.2	69.7	21.0	12.5	14.1
2020-03-24	59.6	38.3	63.5	16.0	11.6	14.7

FEF50 and FEF75 = forced expiratory flow at 50% and 75% of the FVC, FEV1 = forced expiratory volume in one second, FVC = forced vital capacity, MMEF = maximum mid-expiratory flow of forced ventilation, pre = prediction.

## 4 Diagnosis

Whole-exome sequencing was performed using a tri-diagnostic approach (patient and both parents). The results revealed a hemizygous pathogenic mutation c.1010G>A, p. (Arg337Gln) in *FOXP3* (NM_014009.3) (Institute of Birth Cohort, Beijing Children's Hospital, Capital Medical University) in the patient, which was not found in his parents.

## 5 Intervention and outcome

The patient chose oral mycophenolate mofetil (0.5 g/day) with budesonide formoterol inhalation therapy after diagnosis. The patient experienced temporary herpes zoster and aggravated eczematous dermatitis with an unbearable itchy sensation during the treatment period. Although the patient had only 1 episode of mild wheezing in the 6 months of treatment, he eventually discontinued treatment with mycophenolate mofetil and refused other immunosuppressants.

## 6 Discussion

FOXP3 is a member of the forkhead box protein family of transcription factors, and its stable expression is crucial for the development, maturation, and maintenance of CD4+ regulatory Tregs.^[4]^ Functional mutations of *FOXP3* result in a decrease in Tregs or defects and fall within the category of diseases of immune dysregulation.^[5]^*FOXP3* is located in the centromeric region of the X chromosome (Xq11.3-q13.3).^[6]^ Mutations in both coding and non-coding regions can cause IPEX, and the most frequent mutations occur in the FKH domain.^[4]^ Our patient's mutation [c.1010G>A, p. (Arg337Gln)] is located in the common site of the FKH domain and exon 10, but it is sporadic and different from other genealogical cases.^[7–9]^

FOXP3 plays a direct role in suppressing Th2-like Tregs,^[10]^ and the uncontrolled Th2 immune responses of IPEX not only dominate the autoimmune responses in the target tissues, but also hinder the host from mounting effective and appropriate immune responses to invading microorganisms and exogenous antigens,^[11,12]^ which lead to autoimmune diseases, allergies, and recurrent infections. Although the typical clinical manifestations of IPEX are early onset enteropathy, type 1 diabetes mellitus, and skin diseases, almost every system can be involved.^[13–17]^ Our patient's lesions began appearing at six months of age, and were located in the respiratory tract, gastrointestinal tract, and skin, presenting with refractory asthma, recurrent serious respiratory infections, controlled enteropathy, and chronic eczematous dermatitis but without any manifestation of autoimmune endocrinopathy. Therefore, except for male sex, the onset age, affected organs, lesion type, and prognosis can vary, even with the same mutation.^[3,7,9,18–21]^

The patient's respiratory symptoms seemed to be a major problem in the later stages. Lung diseases, including bronchiectasis, emphysema changes, and pulmonary fibrosis, are the most common clinical features of primary immunodeficiency disease.^[22]^ However, lung diseases are not common in patients with typical IPEX,^[4]^ which may be because the lung damage process is a consequence of chronic and recurrent infections paired with inflammatory or autoimmune diseases.^[22]^ Therefore, only patients with atypical and mild disease, like our patient, may develop lung disease. However, it is interesting to note that not all mild cases will cause lung damage,^[9]^ which supports the hypothesis that the epigenetic regulation of FOXP3 expression plays an important role in the development of IPEX. ^[23,24]^ Chronic lung diseases can lead to decreased exercise tolerance, increased fatigue, chronic cough, and oxygen dependence, and significantly impact the patients’ quality of life.

Currently, immunosuppressive therapy is still the first-line therapy for patients with IPEX. Nonspecific immunosuppressive drugs, such as corticosteroids, are often used to control abnormal inflammation during the exacerbation and remission periods. Calcineurin inhibitors such as cyclosporine and tacrolimus,^[25]^ and non-calcineurin inhibitors such as rapamycin, cyclophosphamide, azathioprine, and mycophenolate mofel^[11,26,27]^ are often used to control T cell activation during the remission period. Oral mycophenolate mofetil combined with inhaled budesonide formoterol seemed to control respiratory symptoms well, but induced aggravated eczematous dermatitis with an unbearable itchy sensation, which led the patient to give up treatment. Increasing evidence supports rapamycin, an mTOR inhibitor, as a primary drug (alone or in combination with corticosteroids) because effective T cells are more dependent on the mTOR pathway compared to Tregs.^[1,28]^ However, immune suppression can be effective in improving the symptoms of autoimmune and allergic diseases, but it does not appear to halt disease progression and may induce severe side effects, such as osteoporosis, dyslipidemia, and chronic renal dysfunction.^[27,29]^ The only potentially curative therapy for IPEX syndrome is allogeneic hematopoietic stem cell transplantation (HSCT), and both HLA-identical and matched-unrelated HSCT can be successful.^[1]^ Although cases of diabetes could also be cured by HSCT,^[30]^ the less organ damage, the better the prognosis of HSCT, and partial donor chimerism is enough to relieve symptoms.^[1]^

The clinical manifestations of IPEX are variable and laboratory test results including the number of CD4+ FOXP3+ Tregs,^[31,32]^ serum immunoglobulin levels,^[33,34]^ and types of autoantibodies^[9,35]^ are nonspecific; therefore, we have summarized some diagnostic clues from this case and previous literature:

(1) inflammatory bowel-like disease is 1 of the most prominent overlapping clinical disease features caused by Tregs impairment^[36]^;
(2) early-onset autoimmune diseases^[37]^;
(3) males simultaneously suffers atopic and autoimmune diseases^[12]^;
(4) early-onset inflammatory skin diseases, which rapidly develop and are resistant to strong corticosteroids^[14]^; and
(5) refractory allergic asthma complicated with recurrent serious respiratory tract infections and ventilation function damage.

If the possibility of primary immunodeficiency is considered, genetic testing should be improved as soon as possible.

## 7 Conclusion

The clinical manifestations of IPEX vary. Early diagnosis and treatment are crucial even in cases without endocrine gland injury or those who have atypical presentations and mild courses. Lung injury may be a major problem in the later stages of atypical IPEX, and mycophenolate mofetil seem to be effective for respiratory symptoms, but could induce significant skin side effects.

## Author contributions

**Conceptualization:** Ying Zhang and Hanmin Liu.

**Data curation:** Ying Zhang, Tao Ai, Wanmin Xia and Lei Zhang.

**Formal analysis:** Tao Ai and Hanmin Liu.

**Investigation:** Ying Zhang, Xiulan Luo and Yaping Duan.

**Methodology:** Tao Ai and Hanmin Liu.

**Resources:** Hanmin Liu.

**Writing – original draft:** Ying Zhang, Tingting Chen.

**Writing – review & editing:** Ying Zhang and Hanmin Liu.
